# Construction of an integrated database for hERG blocking small molecules

**DOI:** 10.1371/journal.pone.0199348

**Published:** 2018-07-06

**Authors:** Tomohiro Sato, Hitomi Yuki, Keiji Ogura, Teruki Honma

**Affiliations:** Center for Life Science Technologies, RIKEN, Suehiro-cho, Tsurumi-ku, Yokohama City, Kanagawa, Japan; Pennsylvania State University, UNITED STATES

## Abstract

The inhibition of the hERG potassium channel is closely related to the prolonged QT interval, and thus assessing this risk could greatly facilitate the development of therapeutic compounds and the withdrawal of hazardous marketed drugs. The recent increase in SAR information about hERG inhibitors in public databases has led to many successful applications of machine learning techniques to predict hERG inhibition. However, most of these reports constructed their prediction models based on only one SAR database because the differences in the data format and ontology hindered the integration of the databases. In this study, we curated the hERG-related data in ChEMBL, PubChem, GOSTAR, and hERGCentral, and integrated them into the largest database about hERG inhibition by small molecules. Assessment of structural diversity using Murcko frameworks revealed that the integrated database contains more than twice as many chemical scaffolds for hERG inhibitors than any of the individual databases, and covers 18.2% of the Murcko framework-based chemical space occupied by the compounds in ChEMBL. The database provides the most comprehensive information about hERG inhibitors and will be useful to design safer compounds for drug discovery. The database is freely available at http://drugdesign.riken.jp/hERGdb/.

## Introduction

Blockade of the human ether à-go-go related gene potassium channels is associated with drug-induced QT interval prolongation, which could cause arrhythmia and more severe heart failure [1–3]. The inhibition of hERG has become the major reason for drug withdrawals in the late 1990s, as represented by the withdrawals of terfenadine [4, 5], astemizole [6], and cisapride [7]. Thus, the assessment of the risk of hERG inhibition in the early stages of drug discovery, such as the screening and hit to lead stages, could effectively decrease the cost and failure risk of drug discovery.

Various computational methods to predict hERG inhibition were recently reported [8–23]. These studies included statistical models based on the 2D or 3D structures of small compounds, and structure-based approaches employing docking simulations using a modeled 3D structure of hERG. Although the electronic microscopy structure of hERG was recently solved [24], docking simulations of hERG are still a difficult challenge due to its high flexibility. However, the rapid growth of bioactivity information about hERG in various databases, and the improvement of machine learning techniques have encouraged the use of statistical methods to predict hERG inhibition as summarized by Wang *et al*. [8] and Villoutreix *et al*. [9]. Among recent studies, some prediction models have been built using more than tens of thousands of compounds (for example, 306,895 compounds from hERGCentral database [10], 58,963 compounds from patented data [11]). Despite such studies using so-called big data, most of the previous studies to predict the hERG inhibitory activity are based on fewer than 5,000 compounds, which are often derived from a single database as reported by Villoutreix *et al*. [9]. One major obstacle that limited the amount of hERG-associated data was the differences in the format and ontologies between various databases, which hampered the use of multiple data source.

This study pursued the comprehensive and careful integration of the hERG-associated bioactivity information available in various databases. The hERG-associated data entries were derived from ChEMBL (https://www.ebi.ac.uk/chembl/) [25], GOSTAR (https://www.gostardb.com/index.jsp) [26], NIH Chemical Genomics Center data set registered in PubChem (https://pubchem.ncbi.nlm.nih.gov/bioassay/588834) [27], and hERGCentral (www.hergcentral.org (currently not working)) [28], and then merged by chemical structures after standardization. The procedure included unifications of the data format, value type, units, assay types, and chemical structures, based on the text analysis assisted by visual inspections by medicinal chemistry experts. Although a dataset built from the results of a single assay protocol can make the resulting values consistent and provide high quality data for quantitative analysis, it raises the risk of systematic bias, such as false positive observations in a patch-clamp assay by membrane damaging compounds. Since the integrated dataset contained heterogeneous data entries, the deviations of the hERG inhibitory activities due to the differences in the assay protocols were analyzed, to assess the influence of the deviations on the classification of the tested compounds into hERG inhibitors and non-inhibitors.

The development of high-throughput automated patch clamp assays has increased the amount of hERG-associated data available in public databases. Thus, the time-series increase of the hERG-associated entries was also investigated, using the constructed dataset. The transitions in the number of reported compounds, the number of chemical scaffolds, and the coverage of the chemical space compared to all known biologically active compounds in ChEMBL were assessed. The analysis provides useful insights to interpret the applicability of previously reported statistical models, using different databases. Ultimately, various physicochemical properties were calculated for hERG inhibitors and inactive compounds in the dataset. The results were used to assess the chemical nature of hERG inhibitors, and to determine whether the differences in the data sources affect the statistical analysis or the prediction models built from the data.

## Materials and methods

### Data set

**Fig 1:**
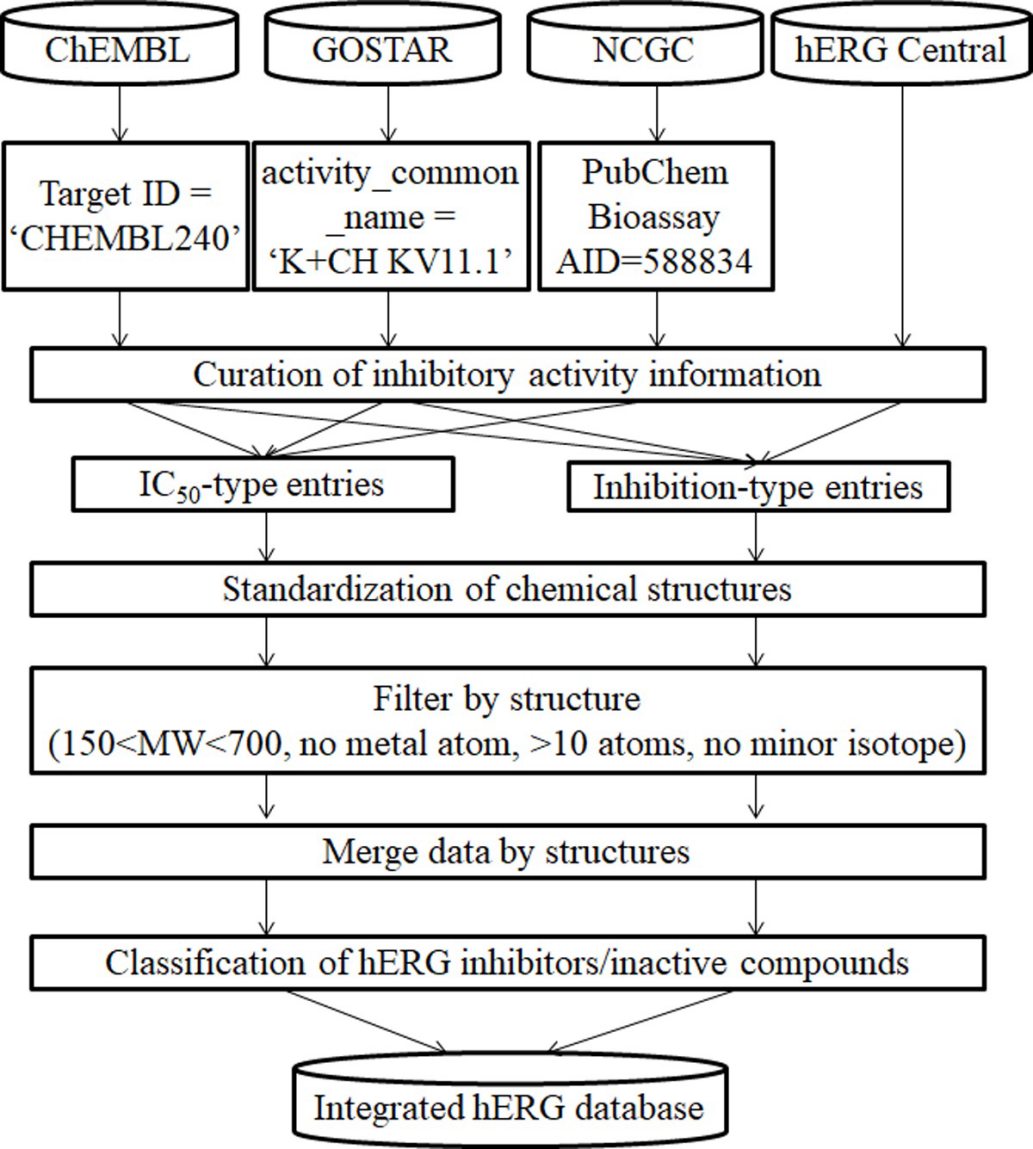
Schematic procedure of the database integration.

The hERG inhibitory activity information was derived from ChEMBL, GOSTAR, NIH Chemical Genomics Center (NCGC) dataset in PubChem bioassay, and hERGCentral, and was integrated as briefly summarized in Fig 1. Procedures for data extraction, formatting activity information, standardization of chemical structures to merge data entries, filtering non-druglike compounds, and classification of hERG inhibitors and inactive compounds, considering the deviation of experimental values are presented in this section. The data set was retrieved from the respective sources in April 2017.

#### ChEMBL

ChEMBL is the bioactivity database maintained by the European Bioinformatics Institute, and is frequently used in various cheminformatics researches as the *de facto* standard database. The entire ChEMBL database (version 22) was downloaded from the web site, and 17,952 activity entries about 2,153 hERG-related bioassays were extracted, according to the Target ID (CHEMBL240) assigned to biological assays. To ensure the validity of the data, entries with data validity comments of “Nonstandard unit for type” and “Outside typical range”, and entries tagged as potential duplicates were excluded. For example, the hERG-related assays reporting selectivity between inhibitors or raw values of tail currents, were removed by investigating their value types and assay descriptions. After manually checking assays reporting more than 20 compounds, several redundant entries that represented the same assay results were removed.

#### GOSTAR

GOSTAR is an online scientific database product of Excelra Knowledge Solutions, consisting of published and patented inhibitors against various biological targets and their associated SAR data. The hERG-associated data entries with activity common name of “K+CH KV11.1” were derived from the GOSTAR database. Although GOSTAR has four subset databases, Drug Database (DD) was excluded in this study, because it includes some critical curation errors during the ontology standardization. The subset will be integrated after the errors are corrected by the vendor. Excluding the 106 entries in the DD subset, GOSTAR contained 14,176 entries about hERG inhibition in the remaining three subsets. Since both ChEMBL and GOSTAR contained hERG activity entries derived from medicinal chemistry journals, the redundant entries with ChEMBL were identified by the reference field and omitted from the data set.

#### NIH Chemical Genomics Center

Quantitative high throughput screening data to determine in vitro hERG channel blockage by NCGC were derived from PubChem bioassays (AID = 588834, v1.1). The data related to hERG, including about 2,688 compounds from the LOPAC1280 library (Sigma), the NTP collection and the NCGC Pharmaceutical Collection (NPC), were determined by FluxORTM thallium flux assays. The dataset contains EC_50_ values for both hERG inhibitors and activators along with some undefined data. As the EC_50_ values contained in the NCGC dataset were calculated from automated sigmoid curve-fitting to the dose responses of hERG activities by the Hill equation, the values had to be interpreted by the Hill coefficient (positive value for hERG inhibitor and negative value for activator) and the fitting quality (low correlation for inconclusive entries). In PubChem, the entries in the NCGC dataset were classified into “Inhibitor”, “Activator”, and “Inactive” in terms of the phenotype. For compound with low quality curve fitting, an “Inconclusive” description was included in the outcome comments. Although the EC_50_ values of the NCGC dataset were also included in the ChEMBL database, the information about the phenotype and outcome comments to interpret the assay results were omitted in ChEMBL. Thus, the corresponding entries were excluded from the ChEMBL dataset. Accordingly, the hERG inhibitors were defined as the entries specified as “Inhibitor” with sufficient inhibitory activity (EC_50_≤10μM in this case) and without outcome comments of “Inconclusive”. All compounds with EC_50_ values exceeding 10μM, were defined as negative compounds in this study.

#### hERGCentral

hERGCentral [28] is a database containing the hERG activity information of more than 300,000 compounds. Since the hERGCentral database (www.hergcentral.org) is currently out of order, the values of the percent inhibitory activities of 318,496 compounds at a 10 μM concentration determined by IonWorks Quattro (MDC, Sunnyvale, CA) in the population patch clamp (PPC) mode were retrieved from the supporting information of a manuscript published by Fang *et al*. [10], describing a statistical analysis of the hERGCentral dataset.

### Formatting activity information

To integrate the hERG activity information collected from the databases, the derived entries were formatted as follows. At first, the entries were classified as either constant concentration values describing inhibitory activity, such as IC_50_, EC_50_, ED_50_, K_i_, K_d_ and percent inhibitory activity at a certain concentration. For convenience, the former data were categorized as IC_50_-type, and the latter were categorized as inhibition-type in this study.

The IC_50_, K_d_, K_i_, EC_50_, and their log-unit values, such as pIC_50_, were obtained from the ChEMBL, GOSTAR, and NCGC data sets for IC_50_-type entries. All values were converted to nM order from their various units. In terms of inhibition-type entries in ChEMBL, GOSTAR, and hERGCentral, entries reported as remaining enzyme activity were converted to inhibition percentage format. For entries in which the assay concentration was not specified, assay descriptions were scanned to complement the compound concentration if possible. Other values, such as concentrations using thresholds other than 50% inhibition (IC_70_, IC_30_, etc.), raw values of measured current, prolonged QT interval ratios, etc., were discarded in this study, because they cannot be directly compared to other entries. Several value types can be used for both hERG activation and inhibitory activities, and thus the assay descriptions and/or reference manuscripts were manually checked for “Kd”, “EC_50_”, “ED_50_”, and “activity” entries to remove the assay entries describing hERG activation.

The assay protocols of hERG blocking activities could roughly classified into electrostatic assays such as automated patch clamp assays that measure the change in the voltage between the cell-membrane by the presence of small molecules, and binding assays, such as radio-ligand replacement assays that measure the binding affinity of small molecules by the replacement ratio of radiolabeled inhibitors. The IC_50_ values and inhibitory percentages generally depend on the assay protocols, and the differences in the hERG assay protocols mentioned above could potentially affect the results, especially in quantitative analyses such as regression models of binding affinity. Using compounds with IC_50_ values determined by both binding assays and electrostatic assays, the correlation between the two methods was investigated. The deviation of the IC_50_ values of each compound was investigated for hERG inhibitors for which more than three IC_50_ values were reported to assess how the deviation of the heterogeneous data affected the characterization of the corresponding compound.

### Standardization of chemical structures

To merge the databases, the chemical structures were standardized for comparison. The structures were obtained from sd files provided by ChEMBL and PubChem (NCGC and hERGCentral) or constructed from the SMILES string (GOSTAR). The obtained structures were desalted, standardized to a neutral ionization state, and then converted to the canonical tautomers using the Pipeline Pilot program. To exclude non-drug like compounds, metal containing molecules, molecules with molecular weights less than 150 or more than 700, molecules with fewer than 10 atoms, and molecules with minor isotopes were removed from the data set. After this structural filtering, the data size of each database was reduced to 14,991 activity entries for 11,993 compounds in ChEMBL, 13,861 activity entries for 8,338 compounds in GOSTAR, 1,894 activity entries for 1,733 compounds in NCGC, and 305,928 activity entries for 303,351 compounds in hERGCentral. These activity entries from the 4 databases were finally merged by the standardized structures.

### Classification of hERG inhibitors and inactive compounds

Using the integrated database, the distributions of the physicochemical properties and the structural diversity of the hERG inhibitors (positive compounds) and the inactive chemicals (negative compounds) were investigated. In this study, positive compounds were defined as hERG inhibitors showing IC_50_ ≤10μM or ≥50% inhibition at 10μM. Since some compounds had multiple assay results, contradictory results due to experimental errors or differences in assay methods were found. As the determination of the IC_50_ value requires measurements of inhibitory activities at multiple concentrations, the IC_50_-type information was considered to be more reliable, and was given higher priority over the inhibition-type entries for positive/negative classification. When the deviation of the assay results was still significantly large among the IC_50_-type entries or inhibition-type entries, the omission of outlier values and majority vote-based procedures was performed to classify hERG inhibitors and inactive compounds as follows.

At first, the compounds for which all assay results unanimously indicated either the existence or absence of sufficient inhibitory activities were assigned to hERG inhibitors or inactive compounds. When contradictory assay values were found, the assay entries that were at least 10-fold higher or lower than the mean value of the compound were defined as outliers, and were removed. Subsequently, a compound was classified as either a positive (hERG inhibitor) or negative (inactive compound), when more than 2/3 of the assay entries of the compound agreed in either category. Qualitative assay results, such as comments describing “no activity” were also included in the voting. Compounds for which comparable numbers of entries indicated opposite results were discarded as inconclusive compounds from the comparison between hERG inhibitors and inactive compounds. The classification procedure was first performed using IC_50_-type entries, and then inhibition-type entries were considered for the remaining unclassified compounds. An investigation of the distribution of the percent inhibition data in hERGCentral revealed that the single concentration results from high-throughput screening (HTS) assays left the low confidence region around the threshold value (50% inhibition at 10μM in this study), as compared to the assay values published in peer-reviewed journals, resulting in some contrary results to IC_50_ values reported in other databases (data not shown). Thus, a more strict threshold (>70% inhibition at 10μM for hERG inhibitors and <30% inhibition at 10μM for inactive compounds) was applied to the inhibition type results from the HTS results in which more than 200 compounds were assayed in a single data source.

### Assessment of structural diversity

To evaluate the usefulness of the integrated database, we assessed the structural diversity of the hERG inhibitors and inactive compounds registered in the database. As the metrics for the structural diversity of the compounds, the number of Murcko framework [29] was counted as reported by Langdon *et al*. [30] and Karawajczy *et al*. [31]. The Murcko framework of a compound was defined as the union of rings and linker atoms connecting them. After the decomposition of all compounds in the dataset as Murcko frameworks, the unique numbers of the derived frameworks were counted for both the hERG inhibitors and inactive compounds. The number of Murcko frameworks was then compared to those generated from the whole ChEMBL database. Since ChEMBL is a bioactivity database for various target proteins, their structural diversity could approximate the chemical space covered by all currently available bioactive compounds.

### Physicochemical properties

Twelve physicochemical properties were computed using Pipeline Pilot, including molecular weight (MW), Ghose-Crippen-Viswanadhan neutral form’s octanol-water partition coefficient (AlogP), octanol-water distribution coefficient with all forms (logD), number of hydrogen bond acceptors (HBA), number of hydrogen bond donors (HBD), number of positively charged atoms when ionized at pH7.4 (N_Cations), number of negatively charged atoms when ionized at pH7.4(N_Anions), molecular surface area (MSA), molecular polar surface area (MPSA), number of rotatable bonds (N_Rot), pKa value of most basic atom (pKa_base), and pKa value of most acidic atom (pKa_acid). This descriptor set based on physicochemical properties is widely used to compare compound data sets in drug discovery [32, 33] and some descriptors that seemed to affect hERG binding, such as pKa, were added according to advice from medicinal chemistry experts. The distributions of the physicochemical properties for both hERG inhibitors and inactive compounds were calculated and compared to assess the characteristic features of hERG inhibitors.

## Results and discussion

### The number of assay records for each compound

**Fig 2:**
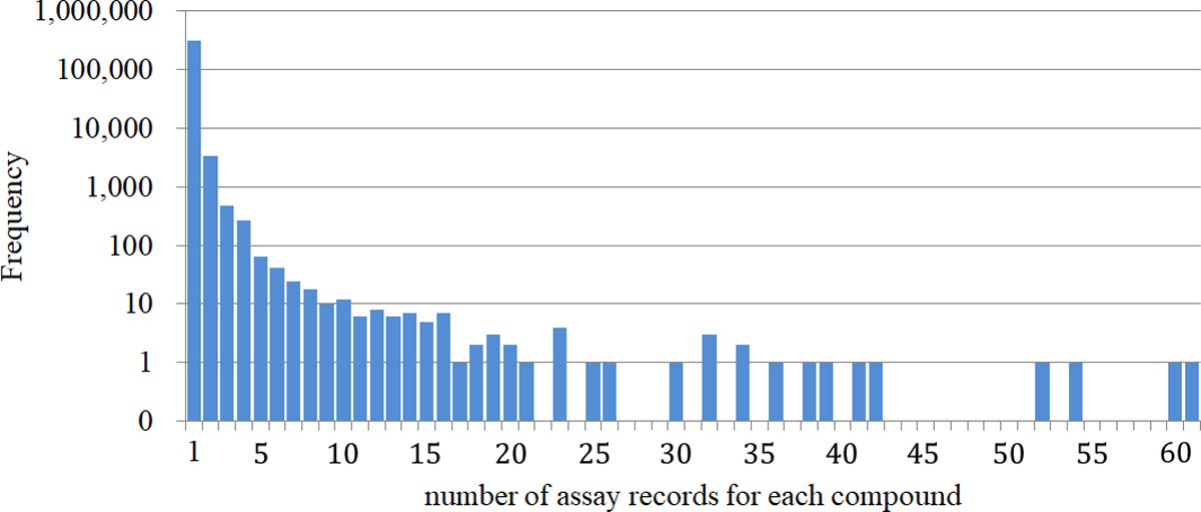
Histogram showing the number of assay records for each compound. The vertical axis representing the frequency is shown in logarithmic scale.

Literature-based bioactivity databases, such as ChEMBL, often contain multiple assay entries for a compound. In the integrated dataset, 329,243 assay records for 319,631 compounds were registered. Using the dataset, the assay records that specified certain values (not using NULL value, ‘>‘, or ‘<‘) for each compound were counted. The distribution of the number of assay records for each compound is shown in Fig 2. The most frequently reported compounds were cisapride (61 assay records), terfenadine (60), dofetilide (54), E-4031 (52), and astemizole (42). While 81 compounds had more than 10 assay records, only one assay record was found for 310,180 compounds mainly reported in the HTS results in the hERGCentral database, resulting in the low average frequency of 1.02 for a compound.

### Comparison between binding assays and electro static assays

**Fig 3:**
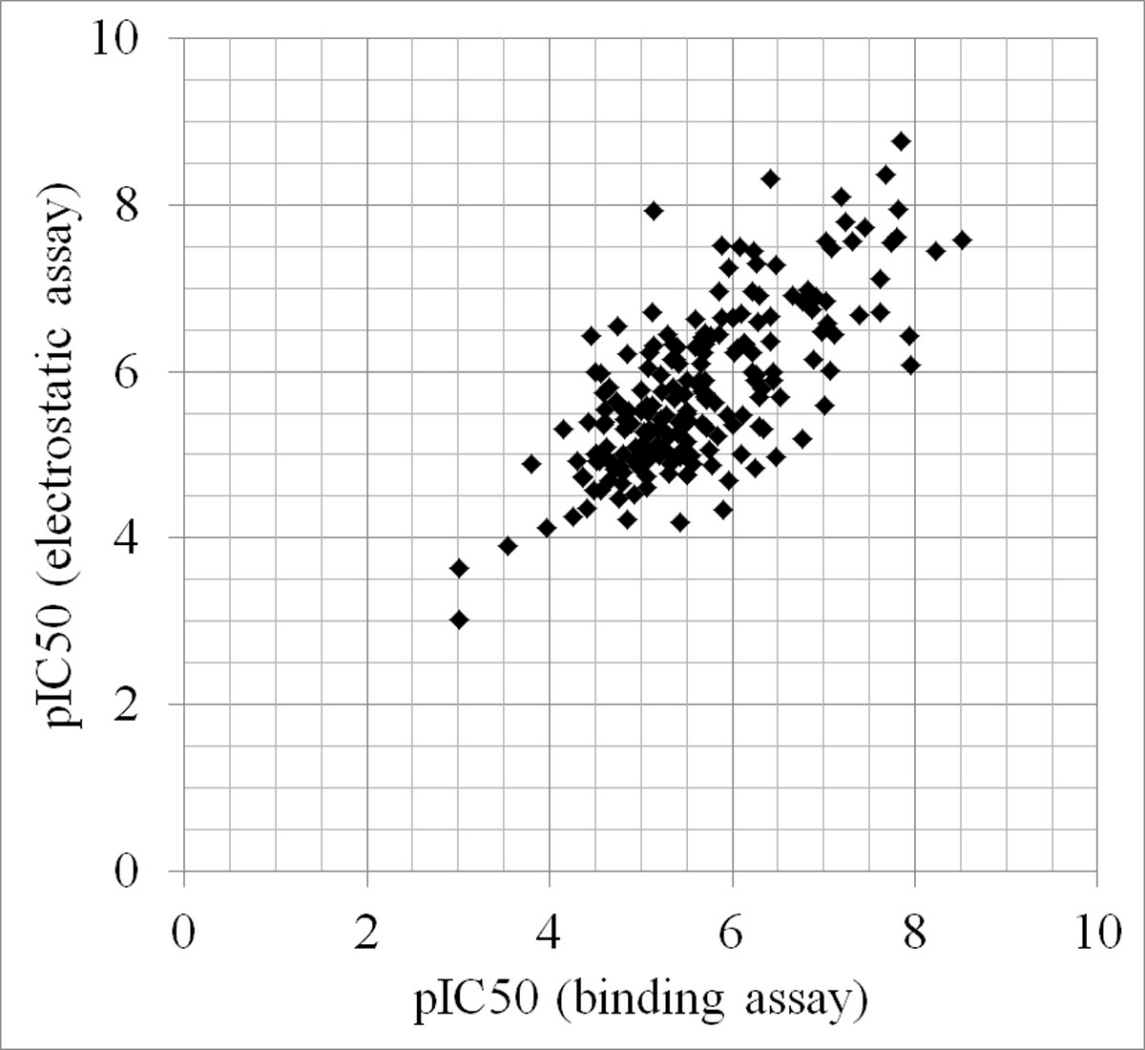
Comparison between mean pIC_50_ values of 209 compounds measured by binding assays and electrostatic assays.

To assess the difference between binding assays such as radio ligand replacement assay and electrostatic assays such as automated patch-clamp assays, the IC_50_ values determined by both methods were investigated using the integrated dataset. In the dataset, 15,932 IC_50_ values were reported for 11,956 compounds. To ensure the validity of the IC_50_ values, 6,594 data entries using inequality signs, NULL values, and improper value range (IC_50_>1mM) were excluded. The remaining dataset contained 4,173 IC_50_ values for 3,449 compounds measured by binding assays, and 3,082 IC_50_ values for 2,246 compounds measured by electrostatic assays. To compare the two methods, 209 compounds for which the IC_50_ values were measured by both methods were investigated. For each compound, the mean pIC_50_ values were respectively calculated for both binding assays and electrostatic assays, and plotted in Fig 3. The averages of the mean IC_50_ values were 2.41μM in binding assays and 1.64μM in electrostatic assays. The coefficient of determination and the root mean square deviation between the pIC_50_ values measured by binding assays and electrostatic assays were 0.517 and 0.737, respectively. While the IC_50_ values determined by both methods showed moderate correlation, slightly higher potencies tended to be observed by electrostatic assays. Among the 209 compounds, 119 compounds showed higher potencies in electrostatic assays. In contrast to binding assays, which directly measure the binding affinity of a compound to the pore region of hERG, electrostatic assays, which measure the decrease of tail-current, could be affected by various additional factors, including non-specific binding to other ion channels, binding to a different region of hERG, or membrane toxicity, possibly resulting in the slightly higher potency observed in the dataset.

### Deviation of IC_50_ values and classification of hERG inhibitors and inactive compounds

**Fig 4:**
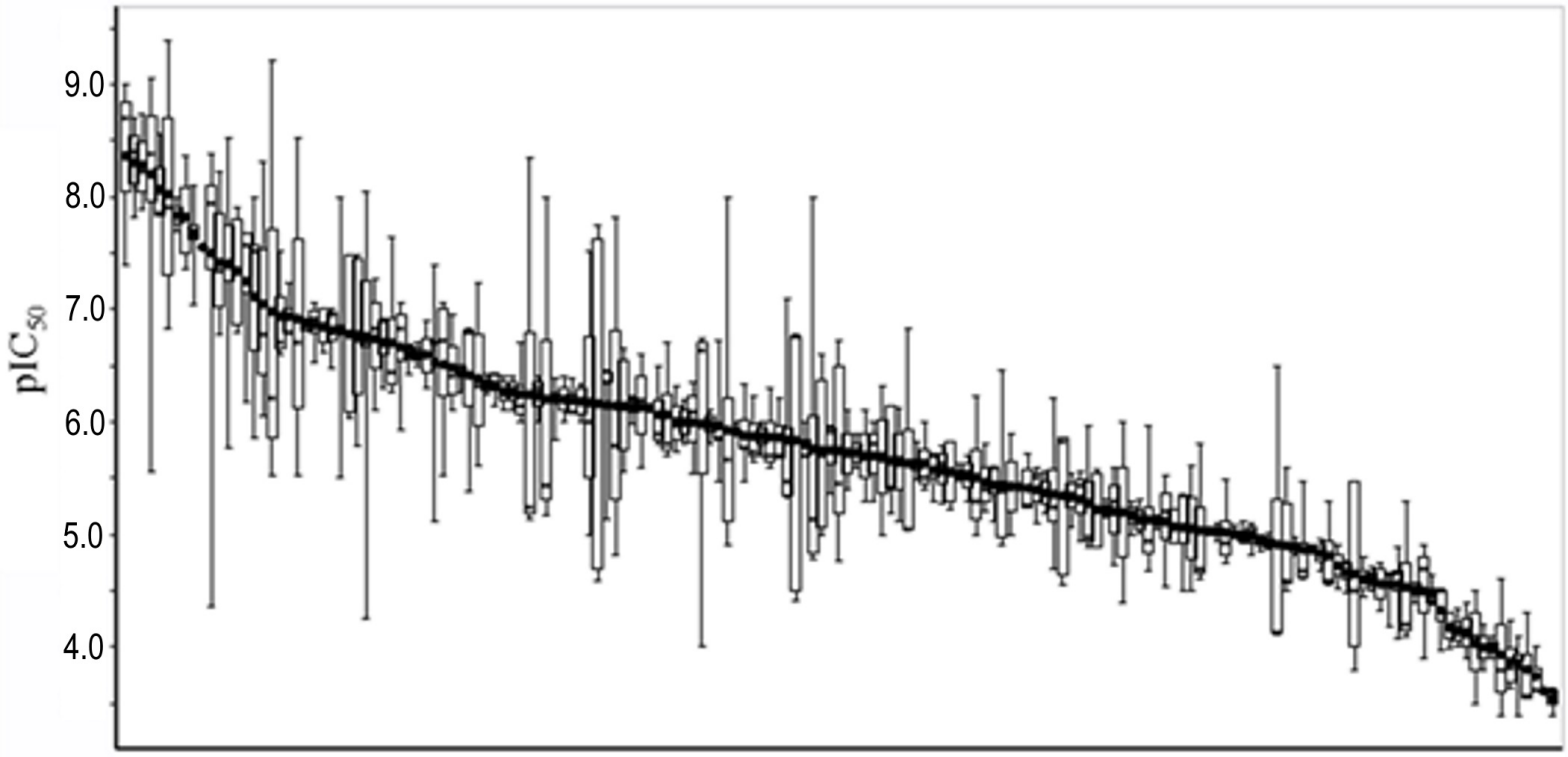
Box plot showing the distribution of IC_50_ values of 263 compounds with more than three reported IC_50_ values. The compounds were sorted by mean values.

In some case, the resulting values had large deviations due to differences in the assay protocols or curation errors such as misinterpretation of units in the manuscript, which could confuse the classification of positive (hERG inhibitors) and negative (hERG inactive) compounds for the statistical analysis. To exemplify the deviation of the hERG inhibitory activities registered in the currently available databases, the distribution of the IC_50_ values of 263 compounds, for which more than three IC_50_ values were reported, is shown in Fig 4. To ensure the validity of the IC_50_ values and exclude extreme values due to curation errors, the IC_50_ values lower than 1pM or higher than 1mM were excluded, because such values were often caused by incorrect registration of the units or misinterpretation of the digits of assay values in the curation procedure.

Among the 263 compounds, 144 compounds showed consistent IC_50_ values with less than one order of magnitude differences between the maximum and minimum results. However, 47 compounds recorded more than 100-fold differences between the maximum and minimum IC_50_ values. An inspection of such cases revealed that only a small number of outlier values often caused such large deviations. Therefore, the differences between the 25th percentile and 75th percentile of IC_50_ values were far less than those between the maximum and minimum values. Only two compounds had more than a 100-fold difference between the 25th percentile and 75th percentile, and those of 224 out of the 263 compounds were lower than 10-fold. These observations emphasize the importance of outlier elimination, to construct a robust dataset and avoid incorrect classification of positive and negative compounds. The integration of various databases increases the number of assay results and enables the detection of more outlier values, as compared to relying on a single database.

**Fig 5:**
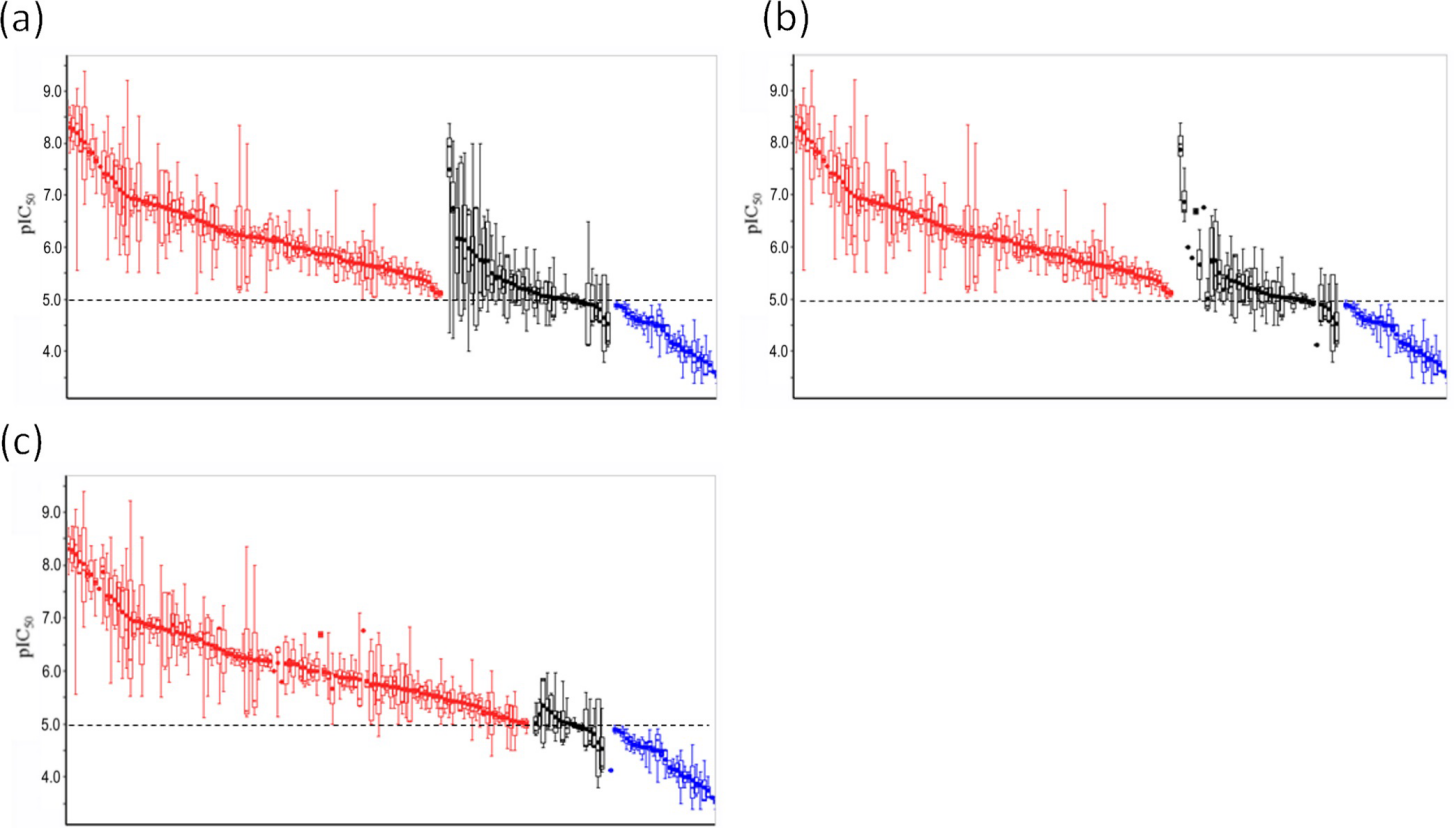
Step-wise procedure for compound classification. (a) Classification by unanimous results using all assay values. Positive, unclassified, and negative compounds are shown in red, black, and blue, respectively. The dashed line indicates the threshold value between positive and negative compounds (IC_50_ = 10μM) (b) Distribution of assay values after removal of outlier values differing more than 10-fold from the mean IC_50_ values from undefined compounds. (c) Classification by a two-thirds majority of filtered IC_50_ values. The remaining unclassified compounds with nearly equal numbers of positive and negative assay results were considered as inconclusive compounds, and omitted from the subsequent analysis of physicochemical properties.

In this study, compounds were classified into either positive (hERG inhibitors showing IC_50_≤10μM or ≥50% inhibition at 10μM) or negative compounds (IC_50_>10μM or <50% inhibition at 10μM). Fig 5 presents the step-wise proceeding of the classification procedure employed to minimize the influence of outlier values and achieve robust classification through unanimous agreement (Fig 5(A)),removal of outlier values (Fig 5(B)), and majority vote using criteria of two-thirds (Fig 5(C)). Considering the large deviation of the IC_50_ values, majority voting was applied to assign the labels rather than using the mean values which could be more sensitive to outlier values. The compounds without IC_50_ information were classified based on the percentage inhibition data. As in the case of the IC_50_ data, assay results showing more than 50% inhibition at a concentration lower than 10μM, and less than 50% inhibition at a concentration higher than 10μM, were counted for each compound, and then assessed according to whether more than two thirds of the data showed consistent results. When both the IC_50_ and percentage inhibition data were available, only the IC_50_ information was considered for the classification because the determination of an IC_50_ value generally requires assays with about 7 different concentrations for sigmoid curve fitting, and thus it could be more reliable than percentage inhibition data measured at a single concentration.

### Structural diversity of the integrated database

Using the standardized structures, the hERG activity entries from ChEMBL, GOSTAR, NCGC, and hERGCentral were merged and classified into hERG inhibitors and inactive compounds according to the aforementioned criteria. Table 1 presents the numbers of compounds in each database and the resulting integrated database, after the removal of inconclusive compounds. The ratios of inhibitors and inactive compounds were nearly 1:1 in ChEMBL and GOSTAR, while they were about 1:6 in NCGC and 1:64 in hERGCentral, respectively. ChEMBL and GOSTAR are literature-based databases from medicinal chemistry journals and patents, and thus they often contain structure activity relationships of compounds sharing certain scaffolds, resulting in a high ratio of hERG inhibitors. The NCGC data set consisted of a pharmacologically active compound library from SIGMA (LOPAC1280 library) and NCGC (NPC). hERGCentral uses the National Institutes of Health (NIH) Molecular Library Small Molecule Repository (MLSMR) as a compound source. Since hERGCentral uses general small molecule library, which is irrelevant to hERG inhibition or other bioactivities, its inhibitor ratio would most likely approximate those in practical HTS in drug discovery project. By merging the compounds from the four databases, a database consisting of 9,890 hERG inhibitors and 281,329 inactive compounds was constructed. The size of the integrated dataset exceeded those of most previous studies. As reported by Villoutreix *et al*. [9], most of the previous studies used datasets consisting of less than 5,000 compounds from public databases, or used in house datasets. The only exception was the study employing 306,895 compounds from hERGCentral dataset by Du *et al*. [10]. The slight decrease of the integrated dataset from original hERGCentral mainly came from the removal of the near 50% inhibition entries in HTS assays in hERGCentral. Because the strict criteria (>70% inhibition at 10uM for HTS assays) was set to remove low confidence entries, the integrated dataset was expected to achieve both the high reliability of assay results and the comprehensiveness of structural diversity.

**Table 1:** The number of compounds and their Murcko frameworks in each database.

Database	Class	Number of compounds	Number of Murcko frameworks
ChEMBL	Inhibitors	4,793	2,474
	Inactives	5,275	3,012
	All	10,068	4,954
GOSTAR	Inhibitors	3,260	1,727
	Inactives	3,509	1,692
	All	6,769	3,098
NCGC	Inhibitors	232	173
	Inactives	1,234	504
	All	1,466	639
hERGCentral	Inhibitors	4,321	2,708
	Inactives	274,536	73,419
	All	278,857	74,687
Integrated database	Inhibitors	9,890	5,516
	Inactives	281,329	76,420
	All	291,219	79,806

The numbers of Murcko frameworks for hERG inhibitors and inactive compounds in each database are shown in Table 1. For hERG inhibitors, the integrated database contains more than twice as many scaffolds than any of the individual databases. Conversely, the structural diversity of the inactive compounds in the integrated database was almost equivalent to that of the hERGCentral dataset. This result revealed the unique nature of the hERGCentral dataset, which contains comprehensive HTS results against large compound libraries covering a broader chemical space than manuscripts or patents focused on specific compounds or chemical series. In total, the compounds in the integrated hERG database contained 79,806 Murcko frameworks. By estimating that the 438,551 Murcko frameworks found in the whole ChEMBL database represent the structural diversity of the all existing bioactive compounds, the integrated database successfully covered 18.2% of that chemical space.

**Fig 6:**
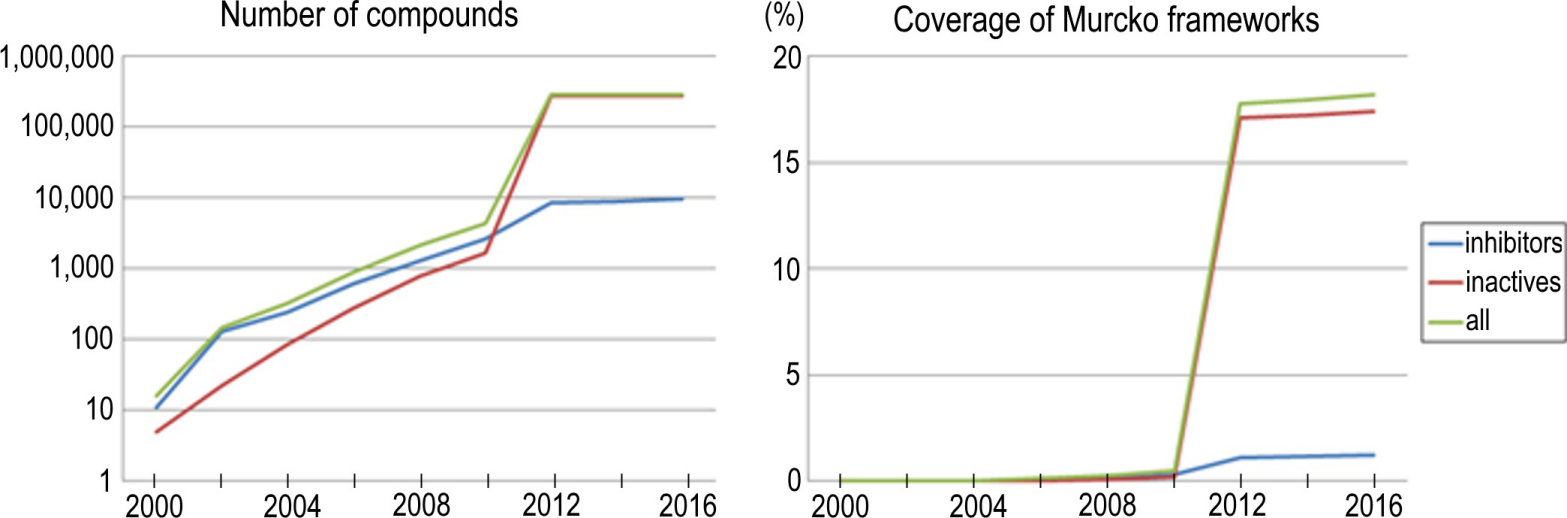
Transition in the number of unique compounds and coverage of Murcko frameworks for hERG inhibitors, inactive compounds, and all reported compounds. Coverage of Murcko frameworks was calculated as the ratio to those of all ChEMBL22 compounds (438,551).

The transition of the number of compounds/scaffolds with hERG activities registered in the databases is shown in Fig 6. Large increases in both hERG inhibitors and inactive compounds were found in 2011, according to the hERGCentral release. Apart from this event, about 200–700 hERG inhibitors and 200–1,000 inactive compounds have been reported each year since 2006. Considering that only 618 hERG inhibitors and 279 inactive compounds were reported in 2006, the recent increase in the hERG-related bioactivity data (covering 18.2% of the Murcko frameworks found in ChEMBL) enables us to construct accurate and robust hERG prediction models for diverse drug-like compounds including newly synthesized ones. The full information about the integrated database at various time points is provided in S1 Table.

### SAR information of each chemical scaffold

**Fig 7:**
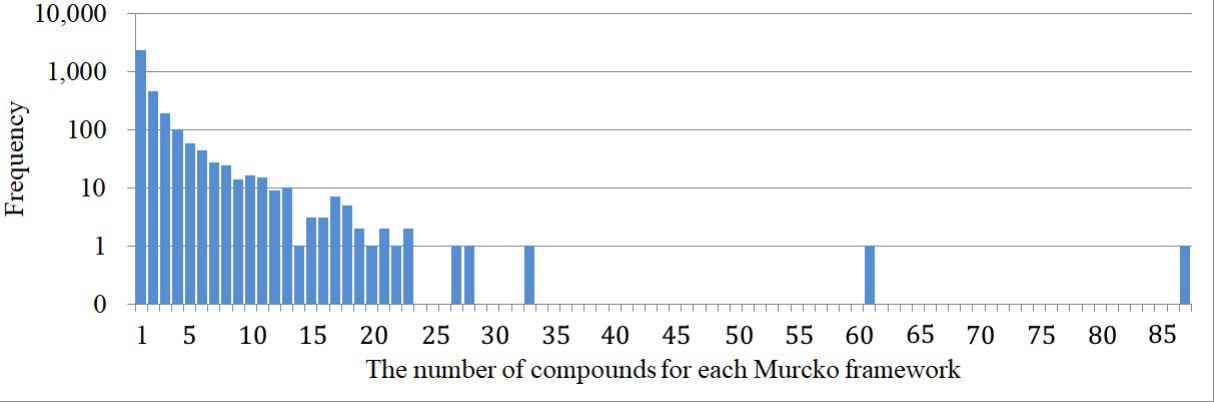
Histogram of the number of compounds in each Murcko frameworks.

**Fig 8:**
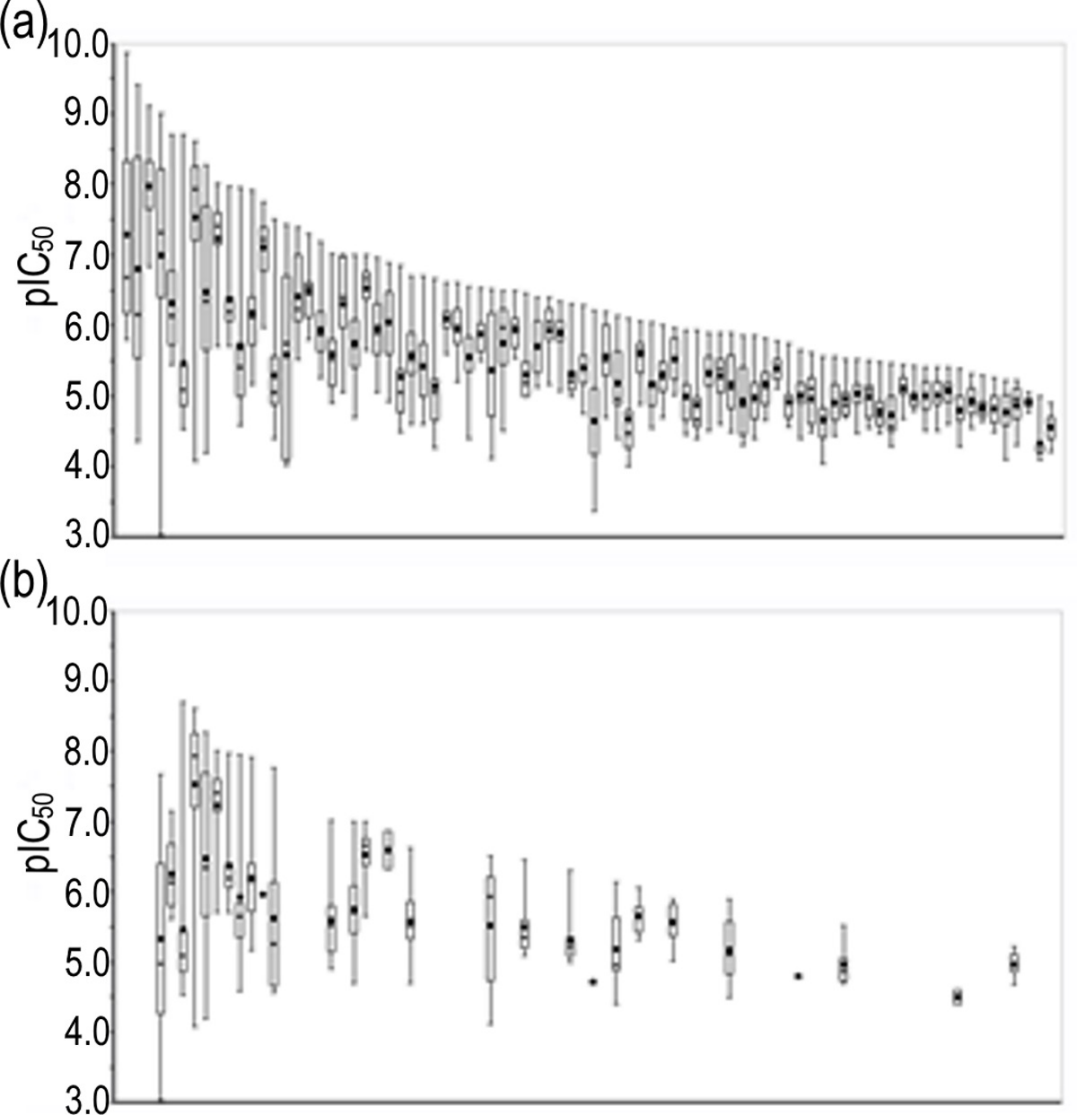
Box plot of IC_50_ distribution about each Murcko framework. (a) IC_50_ values for 82 Murcko frameworks containing more than 10 compounds sorted by the IC_50_ values of the most potent inhibitors. (b) Corresponding IC_50_ values reported before 2009. The horizontal axes of both plots represent each of the 82 Murcko frameworks.

**Fig 9:**
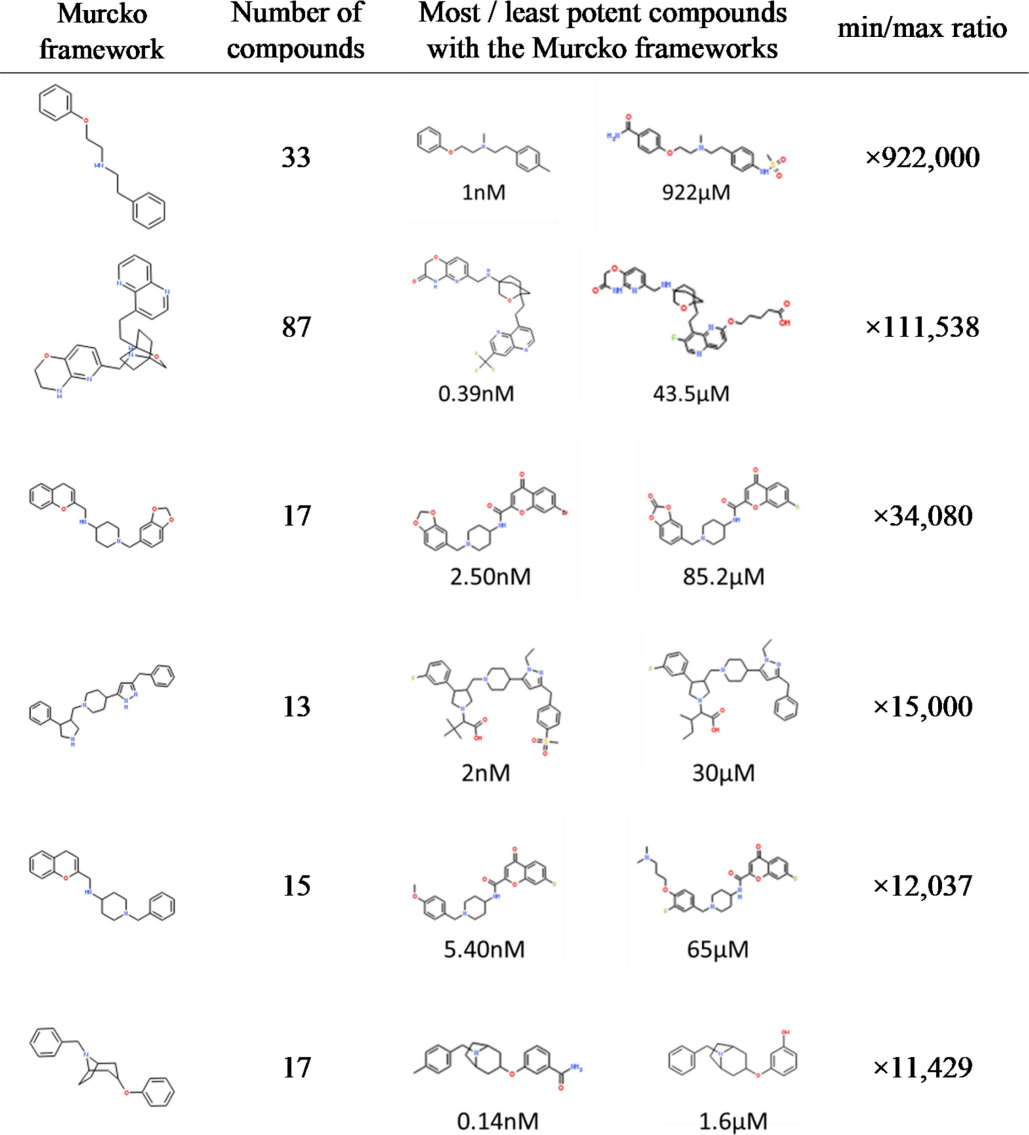
Six Murcko frameworks showing more than 10,000-fold potency differences.

To assess the SAR information of each chemical scaffold contained in the integrated database, the distribution of IC_50_ values among the compounds sharing the same Murcko frameworks was investigated. In the integrated dataset, the IC_50_ values were reported for 3,361 Murcko frameworks. The number of compounds with IC50 entries in each Murcko frameworks is shown in Fig 7. The distribution of IC_50_ values for 82 Murcko frameworks containing more than 10 compounds is presented as a box plot sorted by the IC_50_ value of the most potent compound in each group in Fig 8(A). The mean pIC_50_ value was employed when multiple IC_50_ values were reported for a compound. Six scaffolds showed more than 10,000-fold differences between the IC_50_ values of the most and least potent compounds (Fig 9). All six Murcko frameworks met the requirement of common hERG pharmacophores for charged compounds, consisting of a positively charged atom and two aromatic rings reported in previous studies [34]. Thus, the compounds with various IC_50_ values could provide useful information about the substituents conferring substantial interaction energy with hERG and those decreasing the binding affinity, to design molecules with desirable properties. Such SAR information has also been hugely improved by the recent increase in hERG-related data entries. The IC_50_ distributions of the same chemical scaffolds, reported before 2009, are shown in Fig 8(B). Among the 82 chemical scaffolds, only 28 scaffolds were associated with hERG inhibition before 2009, and several scaffolds lacked highly potent compounds at the time, which could lead to severe bias in the risk assessment of the scaffolds.

### Physicochemical properties

**Fig 10:**
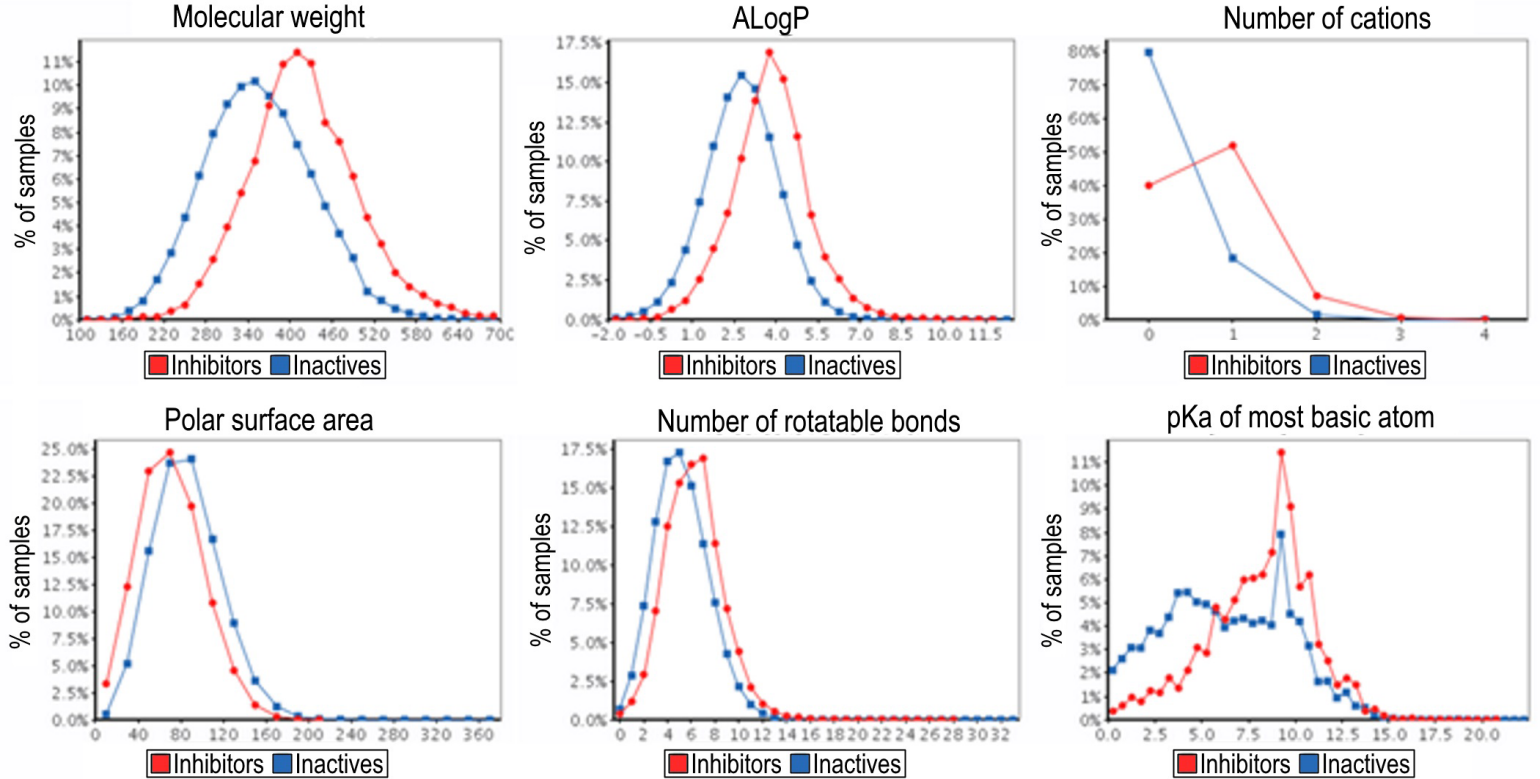
Distribution of 6 physicochemical properties showing significant differences. The distributions of hERG inhibitors and inactive compounds are shown as red and blue lines, respectively.

To assess the contribution of the physicochemical properties to the hERG inhibitory activity, the distributions of 12 physicochemical properties for hERG inhibitors, inactive compounds, and all compounds in the integrated database were compared. The mean and standard deviation of 12 physicochemical properties for both hERG inhibitors and inactive compounds in each database are shown in Table 2. Among the 12 physicochemical properties, the distributions of MW, AlogP, N_Cations, MPSA, N_Rot, and pKa_base, which show significant differences between hERG inhibitors and inactive compounds, are highlighted in Fig 10. The results indicated that hERG inhibitors tend to have a larger molecular weight (418.7, as compared to 355.9 for inactive compounds), higher hydrophobicity according to AlogP and logD (3.80 and 3.22, as compared to 2.77 and 2.58), have more cations (0.68, as compared to 0.22), and less basic substituents (pKa value of most basic substituents was 8.18 as compared to 6.33). While about 80% of the inactive compounds had no positively charged atoms, more than half of the hERG inhibitors contained at least one positively charged atom. These differences were clearly consistent with the previously reported pharmacophores [34] and the expected binding modes of the known hERG inhibitors, because Tyr652 and Phe656 were identified as the important residues frequently forming cation-π and π-π stacking interactions with various hERG inhibitors according to site-directed mutagenesis analyses [35, 36].

**Table 2:** Mean values (standard deviations) of 12 physicochemical properties for hERG inhibitors, inactive compounds, and all compounds in the integrated database.

Property	Inhibitors	Inactives	All
MW	418.7(77.1)	355.9(76.6)	358.0(77.5)
AlogP	3.80(1.39)	2.77(1.34)	2.80(1.35)
logD	3.22(1.47)	2.58(1.41)	2.60(1.42)
HBA	4.47(1.67)	4.41(1.64)	4.41(1.64)
HBD	1.07(0.94)	1.25(0.91)	1.24(0.91)
N_Cations	0.68(0.63)	0.22(0.46)	0.23(0.47)
N_Anions	0.03(0.17)	0.11(0.33)	0.11(0.33)
MSA	424.7(75.7)	356.8(75.0)	359.1(76.0)
MPSA	71.0(30.6)	86.1(31.6)	85.6(31.7)
N_Rot	6.24(2.43)	5.22(2.32)	5.25(2.33)
pKa_base	8.18(2.74)	6.33(3.31)	6.41(3.31)
pKa_acid	3.33(4.15)	2.67(3.71)	2.69(3.73)

Although the aforementioned general trends between hERG inhibitors and inactive compounds were commonly observed in each database, slightly different property distributions were found across the databases (S1 Table). ChEMBL and GOSTAR, which are based on scientific journals and patents, often contain hERG inhibitors and structurally similar derivatives synthesized from an initial hit compound. The mean MWs were 438.7 for hERG inhibitors and 417.0 for inactive compounds in ChEMBL, and 432.7 for hERG inhibitors and 417.8 for inactive compounds in GOSTAR. In contrast, the NCGC dataset, consisting of the LOPAC1280 library, contained much smaller and more hydrophilic compounds as compared to ChEMBL and GOSTAR. The mean MWs were 345.1 for inhibitors and 273.1 for inactives; and for AlogP they were 3.75 for inhibitors and 1.87 for inactives on average. The size of the compounds in the hERGCentral dataset fell between them, with mean MWs of 399.2 for inhibitors and 354.5 for inactive compounds. The difference in the molecular sizes could be due to the fact that the compounds published in medicinal chemistry journals or patents were often synthetically optimized to achieve high potency or desirable properties, and this process generally increased their size from the initial hit compounds in chemical libraries for HTS. Thus, the construction of hERG prediction models based on only a single database could be severely affected by the choice of the database. These results emphasize the importance of the collection and integration of SAR information from various databases, to cover a wider chemical space and enable a more robust analysis. The full description of the physicochemical properties for each database is available in S1 Table.

## Conclusion

By integrating the ChEMBL, GOSTAR, NCGC, and hERGCentral datasets, the largest dataset for the hERG blockade activities of small compounds was constructed in this study. The assay deviation of each compound revealed that large deviations up to more than 100-fold could occur by incorrect curation, emphasizing the importance of removing the outlier values. After the consideration of multiple assay entries, the dataset consisting 9,890 hERG inhibitors and 281,329 inactive compounds was built. The database covered 18.2% of all of the Murcko framework-based chemical space occupied by ChEMBL compounds. The amount of hERG activity information has dramatically increased over the past decade. The number of reported hERG inhibitors increased almost 10-fold in the past 10 years, along with about a 100-fold increase of inactive compounds. The variety of chemical scaffolds commonly (more than 10 compounds) found in hERG inhibitors also increased nearly 3-fold (only 28 scaffolds were reported before 2009, among the 82 scaffolds currently reported).

Investigations of the physicochemical properties of hERG inhibitors in the integrated database reproduced their well-known characteristics. hERG inhibitors tend to exhibit larger molecular weight, more hydrophobicity, more cationic atoms, and less polar surface area. A notable observation was found in the difference of the property distribution among the individual databases. The molecular weight distribution in each database reflected the origin of the compounds where the database collected the SAR information. These differences in the data sources could affect the interpretation of the physicochemical properties in the construction of prediction models for hERG inhibition when the prediction model is built using only one data source. The integration of hERG-associated information from various databases would decrease the bias of the data sources and provide a robust data set for statistical analysis.

The integrated database is available at our home page (http://drugdesign.riken.jp/hERGdb/), with the exception of GOSTAR, which is a commercial database that is not publicly accessible. The current interface allows structural searches and filtering by assay types or data sources. More features such as keyword search, ftp downloads, etc. are planned for future development. The authors are currently constructing a discrimination model of hERG inhibitors and inactive compounds based on the integrated database, which has already showed promising prediction performance exceeding those by commercial software to predict hERG inhibition. The prediction model will be released publicly with the integrated database itself on our homepage. Along with the functional additions, updates of the integrated database to accommodate to latest versions of the ChEMBL and NCGC datasets are ongoing. The corresponding changes in the basic statistics of the updated database and the distribution of molecular properties will be presented on the home page.

## Supporting information

S1 Table Statistics of the physicochemical properties for both hERG inhibitors and inactive compounds in each individual database and the integrated database at various time points. (XLS)
